# Antimicrobial use on 74 Japanese pig farms in 2019: A comparison of Japanese and European defined daily doses in the field

**DOI:** 10.1371/journal.pone.0255632

**Published:** 2021-08-06

**Authors:** Kyoko Fujimoto, Mai Kawasaki, Yuko Endo, Takashi Yokoyama, Itsuro Yamane, Hisanori Yamazaki, Katsumasa Kure, Takeshi Haga, Katsuaki Sugiura

**Affiliations:** 1 Laboratory of Environmental Sciences for Sustainable Development, Graduate School of Agricultural and Life Sciences, The University of Tokyo, Bunkyo-ku, Tokyo, Japan; 2 Division of Bacterial and Parasitic Disease, National Institute of Animal Health, National Agriculture and Food Research Organization, Tsukuba-shi, Ibaraki, Japan; 3 Value Farm Consulting, Tsukuba, Ibaraki, Japan; 4 Department of Veterinary Medical Sciences, Graduate School of Agricultural and Life Sciences, The University of Tokyo, Bunkyo-ku, Tokyo, Japan; 5 Nippon Institute for Biological Science, Ome, Tokyo, Japan; University of Lincoln, UNITED KINGDOM

## Abstract

Defined daily doses (DDD) have been established in human medicine to standardize the measurement of treatment in a population. In veterinary medicine, the European Medicine Agency published defined daily dose (DDDvet) values for antimicrobial agents used in food-producing animals in 2016. National defined doses (DDDjp) for antimicrobials used for pigs in Japan have recently been determined. The aim of this study was to compare the results of calculated antimicrobial use in the field using the DDDjp and DDDvet values. Data from 74 pig farms in Japan relative to antimicrobial use in 2019 was collected. The numbers of DDDs (the weight of biomass treated in kg-days) using DDDjp and DDDvet values for each farm and for different antimicrobial classes were compared. Associations between calculated numbers of DDDjp and DDDvet on farm level were investigated. In addition, differences in antimicrobial use were investigated between different production types of farms (farrowing, finishing and farrow-to-finish farms). Using DDDjp and DDDvet values, the aggregated number of DDDs for 74 farms were 4,099,188 and 2,217,085 respectively, with the former being larger by 1.85 times than the latter. The most frequently used antimicrobial class was penicillin regardless of whether DDDjp or DDDvet was used. The absence of DDDvet values for certain antimicrobial agents used in Japan and the differences in the number of DDDjps/PCU and DDDvets/PCU indicated the need for Japanese DDDs. The number of DDDs per kg population correction unit (PCU) per farm tended to be higher in farrowing farms than in farrow-to-finish farms and finishing farms, with no significant difference (*P* = 0.19).

## Introduction

Antimicrobial resistance is a global one health challenge. There is evidence that antimicrobial use in animals plays a role in the emergence and dissemination of resistant bacteria [1–3]. Currently, 700,000 people die of resistant infections every year in the world. If no proactive solutions are taken to reduce the rise of drug resistance, 10 million lives per year could be at risk from drug resistant infections by 2050 [4]. Development and transmission of bacterial resistance is complex: it arises by mutation and selection, or by acquiring the genetic information that encodes resistance from other bacteria [5]. The loss of efficacy of antimicrobials due to the presence of resistant bacteria, as seen in human medicine, also arises in veterinary medicine. Consequently, prudent use of antimicrobials to prevent antimicrobial resistance is not only important from a public health perspective, but also for animal health and welfare. Moreover, there is evidence of transmission of resistance between animals and humans: The resistant bacteria can be transmitted from animals to humans via direct contact between animals and humans, or through the food chain and the environment. Antimicrobial-resistant infections in humans can cause longer illnesses, increased frequency of hospitalization, and treatment failures that can result in death [6]. As is the case in many European countries, over half of the veterinary antimicrobials purchased in Japan is used in pigs [7–9]. Therefore, reducing the use of antimicrobials and the promotion of a prudent use in pig production are important to reduce selection pressure and thus to lower the resistance rate.

Much work is currently underway to reach a global consensus on antimicrobial use data collection and reporting methods [10,11]. Under the European Surveillance for Veterinary Antimicrobial Consumption (ESVAC) project of the European Medicine Agency (EMA), 31 EU member countries reported total quantities of antimicrobials sold in food animals as mg of active ingredient, adjusted by animal biomass (population correction unit: PCU) in 2018 [9]. The World Organisation for Animal Health (OIE: Office International des Epizooties) is attempting to develop a data collection system that enables the monitoring of antimicrobial use in each member country using a similar metric (mg of active ingredient per kg of animal biomass) [12]. However, the disadvantage of using these metrics is that the different potencies of different antimicrobial agents are not taken into account [13].

In Denmark, the Netherlands and some other European countries and Canada, dosage-based indicators are used to monitor antimicrobial use at the farm level [11,14,15]. Using dose-based indicators, dosage differences between active ingredients and formulations can be corrected and developments can be measured over time, despite changes in the active ingredients used [16]. In 2016, the EMA published average defined daily dose (DDDvet) values for antimicrobial agents used in food-producing animals as a tool to facilitate standardized collection and presentation of AMU among EU members [17]. These values were defined by calculating the mean of dosages of antimicrobial products registered in nine different EU member states. In analogy with the principles of the EMA [18], we have recently assigned national defined daily doses (DDDjp) for antimicrobial agents used in pigs in Japan and verified the need of DDDjp values by comparing them with corresponding DDDvet values [19].

This study takes one more step forward to verify this by applying DDDjp values at farm level. This study aims to investigate the outcome of quantified antimicrobial use at farm level in terms of the total weight of active ingredient and the number of DDDs using Japanese defined doses and European values (DDDvet). The effects of using either DDDjp or DDDvet values were tested for different administration routes and antimicrobial classes. Moreover, the impact of using either DDDjp or DDDvet for quantification of antimicrobial use at farm level on the study farms was considered.

## Materials and methods

### Pig production in Japan and selection of pig farms included in this study

**Fig 1:**
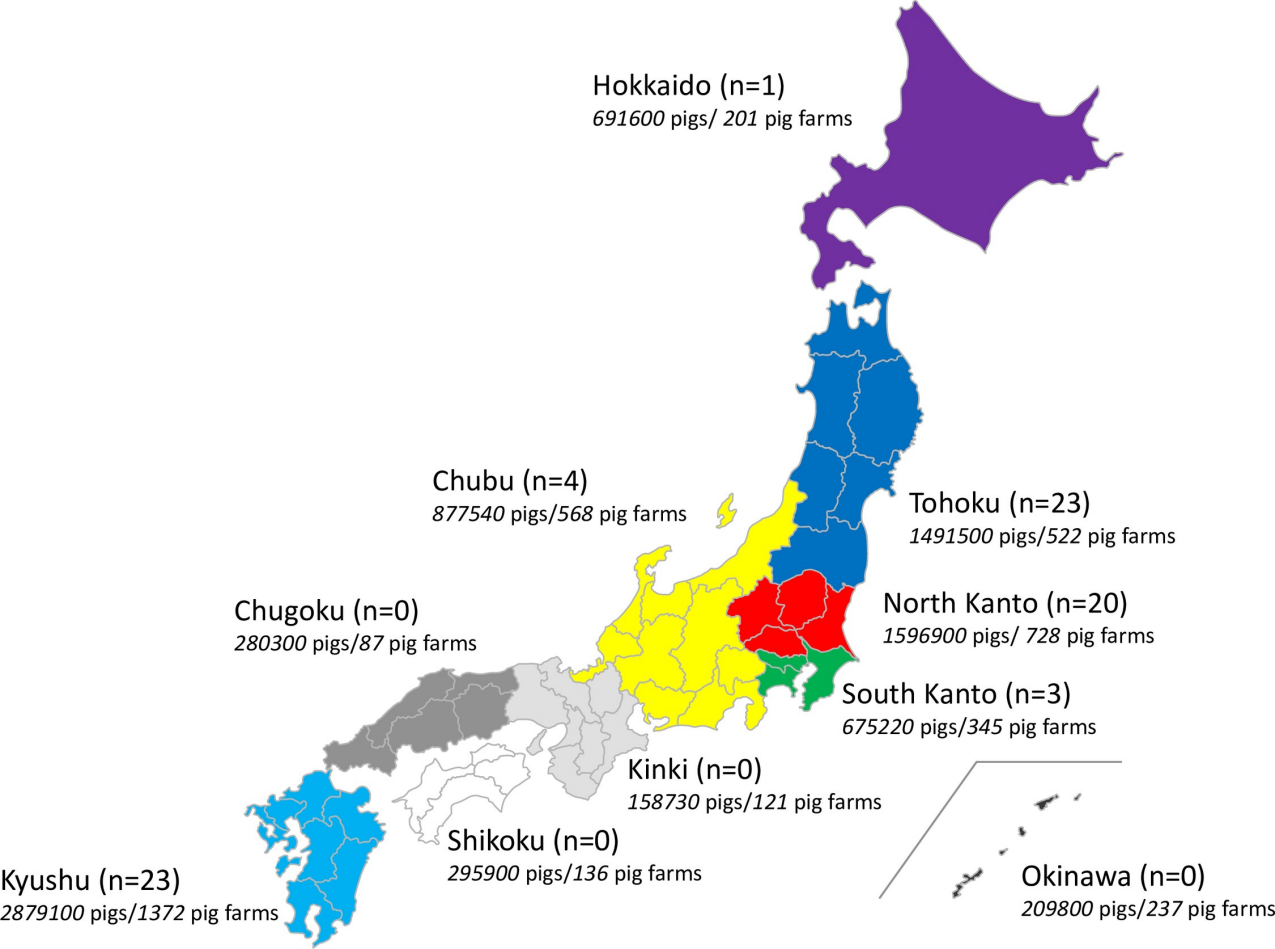
Locations of 74 pig farms analyzed in this study. Numbers in italics indicate the number of pigs and pig farms in the respective regions of Japan as of 1 February 2019. Blank map used for preparation of this figure was reprinted from https://www.freemap.jp/ under a CC BY license, with permission from Keisuke Inoue, original copyright 2006.

As of February 2019, there are 4,320 pig farms keeping 9,156,000 pigs in Japan [20]. Kyushu and Kanto are the two major pig production regions with 2,879,100 and 2,272,120 pigs (31.4% and 24.8%) respectively, followed by the Tohoku region (16.3%) and the Chubu region (7.3%). Most (86.5%) of the farms are farrow-to-finish farms, followed by finishing farms (9.0%) and farrowing farms (4.6%) [21]. Farrow-to-finish farms and farrowing farms have an average of 247 sows. Of the pig farms in Japan, around 1,000 farms are estimated to be in contract with veterinarians from the Japanese Association of Swine Veterinarians (JASV). Of these farms, 87 submitted antimicrobial use data under the PigINFO Bio program from 1 January to 31 December 2019. The authors obtained the data analyzed in this study from PigINFO Bio database anonymously according to the agreement of 1 April 2020 between the Ministry of Agriculture, Forestry and Fisheries and National Agriculture and Food Research Organizations. In accordance with this agreement the JASV veterinarians collected antimicrobial use and other relevant data from pig farmers with verbal consent. PigINFO is a benchmarking system that monitors the productivity of pig farmers, developed jointly by the JASV and the National Agriculture and Food Research Organizations [22]. PigINFO Bio is a part of PigINFO introduced in 2016 to monitor antimicrobial use of pig farmers. Of these farms, 74 farms for which the annual number of pigs shipped for slaughter or fattening from 1 January to 31 December 2019 was available were analyzed in this study. The geographic locations and farm type of these 74 farms are shown in Fig 1 and Table 1. Of these 74 farms, 15 were farrowing farms, 3 were finishing farms and 56 were farrow-to-finish farms. Those farrowing farms keep piglets until they are shipped for fattening at a weight of approximately 35kg; less than 2% of the piglets are retained as replacement gilts.

**Table 1:** Geographical distribution and distribution by farm type of the 74 pig farms subjected to the analysis in this study.

	Hokkaido	Tohoku	North-Kanto	South-Kanto	Chubu	Kinki	Chugoku	Shikoku	Kyushu	Okinawa	Total
Farrowing farm	0	2	0	0	0	0	0	0	13	0	15
Finishing farm	0	2	0	0	0	0	0	0	1	0	3
Farrow-to-finish farm	1	19	20	3	4	0	0	0	9	0	56
Total	1	23	20	3	4	0	0	0	23	0	74

### Collection of antimicrobial use data

Prescription data issued to the study farms from1 January to 31 December 2019 were collected via JASV Veterinarians in contract with these farms, sent to the PigINFO secretariat and entered into the PigINFOBio database. Farms that purchased antibacterial products with prescriptions issued by non JASV veterinarians submitted delivery record data to PigINFO secretariat. These data were sent mostly electronically and partly in paper format. The weight of active ingredient for each antimicrobial agent was calculated based on the name of antimicrobial products and the number of their packages stated in the prescriptions or delivery records. We did not make distinction between feed and water administrations because some of the antimicrobial products were applicable for both and no information was available from the prescription or delivery record as to which of the two routes of administration was applied. In addition to injectable and oral products, there is one intranasal product approved for use in pigs in Japan (kanamycin for prevention of atrophic rhinitis), whose use was negligible therefore was not included in this study.

### Calculation of the weight of active ingredient

To calculate the weight in grams of active ingredient administered, we used a seven-digit ID coding system for antimicrobial products developed by Matsuda et al. (2018) based on the World Health Organization (WHO) Anatomical Therapeutic Chemical Veterinary (ATCvet) classification system (https://www.whocc.no/atcvet/) [23]. In this coding system, the seven-digit code is used as a unique identifier for each antimicrobial package size, dosage and formulation of the antimicrobial presentation. The unique seven-digit code identifies which antimicrobial class (of the total of 13 classes) the product is classified into and antimicrobial active ingredient (a total of 42 active ingredients) the product contains and the administration route (injection or oral) used. The 13 antimicrobial classes include the following: tetracyclines, amphenicols, penicillins, cephalosporins, sulfonamides, trimethoprim, macrolides, lincosamides, aminoglycosides, quinolones, polymyxins, pleuromutilins and others. The specific classification of antimicrobials used on the studied farms is presented in S1 Table. In converting international units (IU) to the weight in grams of active ingredient (e.g. benzylpenicillin) and from prodrug content to active ingredient (e.g. from procaine penicillin to penicillin), the same conversion factors as used by ESVAC were employed [17,18]. Use of penicillins was quantified for different subclasses as well (beta-lactamase sensitive penicillins and penicillins with extended spectrum).

### Calculation of the number of defined daily doses

To calculate the number of DDDjps and DDDvets of each antimicrobial active ingredient, the weight of prescribed antimicrobial ingredient during 2019 of all participating farms was divided by the defined daily doses (DDDjp and DDDvet) of the corresponding antimicrobial ingredient (S1 Table):

$$\text{Number}\;\text{of}\;\text{DDDs}\;\text{of}\;\text{an}\;\text{antimicrobial}\;\text{ingredient}\;a=\frac{\text{Weight}\;\text{of}\;\text{prescribed}\;\text{antimicrobial}\;\text{ingredient}\;a}{\text{DDD}\;\text{value}\;\text{of}\;\text{antimicrobial}\;\text{ingredient}\;a}$$

The DDDvet values were available from the EMA website [17]. The detailed procedure for defining the national doses and all DDDjp values are described in a previous report [19]. To calculate the number of DDDvets for those antimicrobial ingredients for which DDDvet is not available, namely sulfamonomethoxine (for injection), tulathromycin (for injection), dihydrostreptomycin (used in combination product for injection), chlortetracycline(used in combination product for injection), sulfamonomethoxine (for oral administration), streptomycin (used in combination product for oral administration), DDDjp value was used instead.

The amount of active ingredient and the corresponding number of DDDjps and DDDvets were calculated for the different administration routes (injection and oral) and for all antimicrobial classes.

### Indicator used to measure antimicrobial use on each pig farm

The annual quantities of antimicrobials used on a farm in mg of active ingredient per kg of PCU (mg/kg PCU), number of DDDjps per kg of PCU (DDDjps/kgPCU) and number of DDDvets per kg of PCU (DDDvets/kgPCU) were used as an indicator to measure antimicrobial use at farm level. The amount of PCU for each farm was calculated as the sum of biomass of sows and biomass of piglets and fattening pigs shipped for slaughter or fattening during the year 2019. Standard weights of 240 kg and 65 kg were used for sows and slaughter pigs, respectively, as proposed by ESVAC [9]. The standard treatment weight of 17.5kg was used for piglets shipped for fattening based on the fact that they are marketed at an average weight of 35kg in Japan [24]. Of the 74 farms analysed in this study, there were 21 farms for which the data on the number of sows was not available. For these farms, the number of sows was estimated by dividing the sum of the number of slaughter pigs and the number of piglets retained as replacement gilts by 22.9, which is the average annual number of piglets per sow in Japan [25].

### Statistical analysis

The relationship between Japanese and European dosages was evaluated using scatterplots and correlation analysis performed by Spearman’s Rho test. The difference in antimicrobial use between different types of farms (farrowing, finishing and farrow-to-finish farms) was investigated using the Kruskal-Wallis test for independent samples after testing for normality using the Shapiro-Wilk test. Statistical analysis was conducted using Excel 2010 (Microsoft Corporation) and BellCurve for Excel ver. 3.00 (Social Survey Research Information Co., Ltd.) added into Excel.

## Results

### Antimicrobial use quantification per administration route

**Fig 2:**
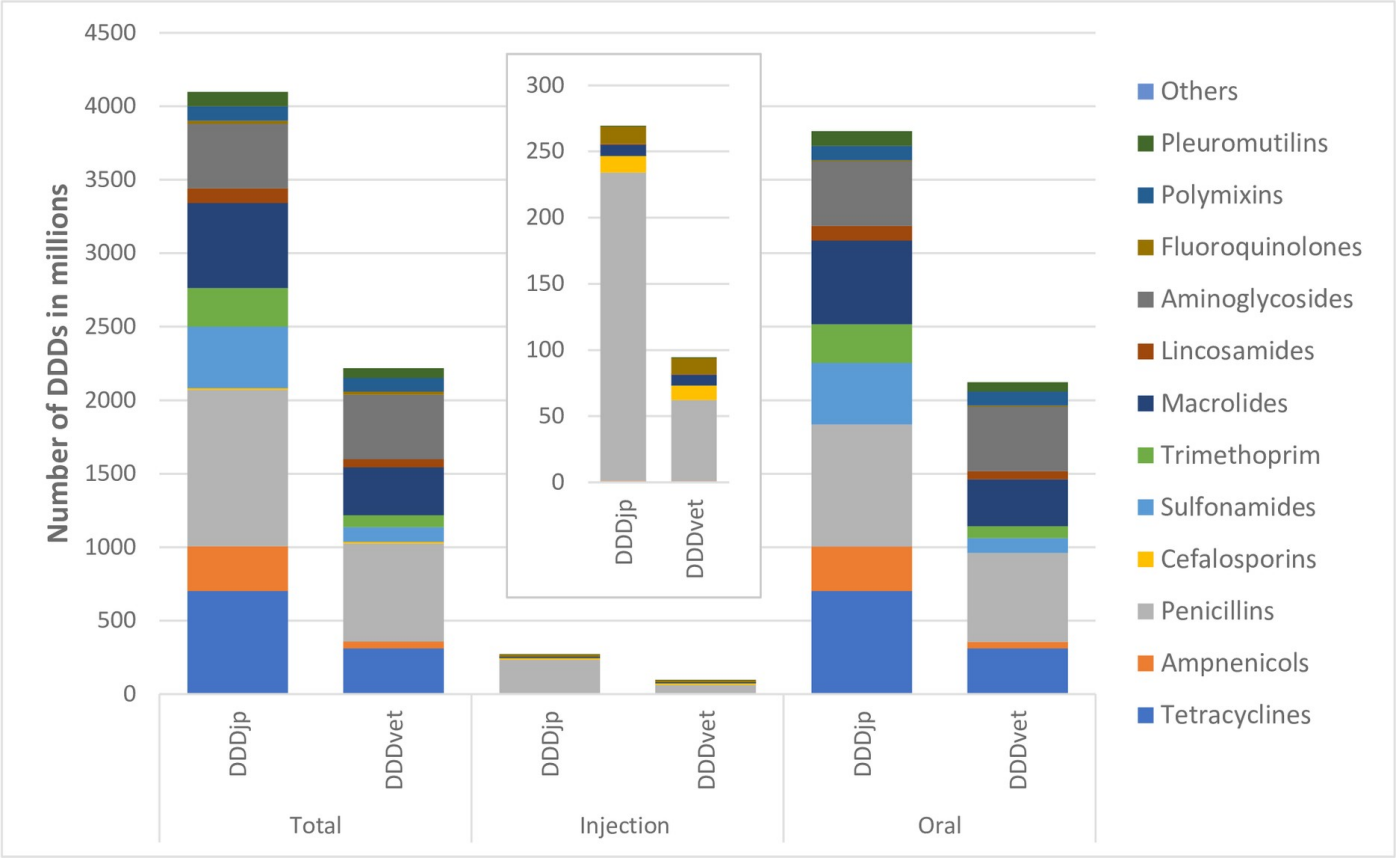
Comparison of the number of defined daily doses (DDD) of antimicrobial use on the 74 pig farms analysed in this study calculated using Japanese values (DDDjp) and European values (DDDvet) published by the European Medicine Agency (EMA).

The aggregated AMU for the 74 pig farms was calculated to be 20,526,121 g of active ingredients and 4,099,188 DDDjps and 2,217,085 DDDvets using Japanese DDD values and European DDD values respectively. The number of DDDs was greater when calculated using DDDjp than when calculated using DDDvet: by 1.85 times for total, and 2.85 and 1.80 times for injectable and oral administrations respectively (Table 2 and Fig 2). When investigating the different administration routes by the number of DDDs, the number of DDDs by oral route represented the larger proportion (93.4–95.9%) regardless of whether the DDDjp or DDDvet values were used (Table 2).

**Table 2:** Total and relative antimicrobial uses on the 74 Japanese pig farms in the year 2019.

Administration route	Amount of active ingredient in g		Number of DDDjp (in 1000)		Number of DDDvet (in 1000)		Ratio*
Injection	840,176	4.1%	269,391	6.6%	94,383	4.3%	185%
Oral	19,685,946	95.9%	3,829,797	93.4%	2,122,702	95.7%	80%
Total	20,526,121	100.0%	4,099,188	100.0%	2,217,085	100.0%	85%

*: Ratio was calculated by (number of DDDjp—number of DDDvet)/number of DDDvet x 100.

### Antimicrobial use quantification per antimicrobial classes

**Fig 3:**
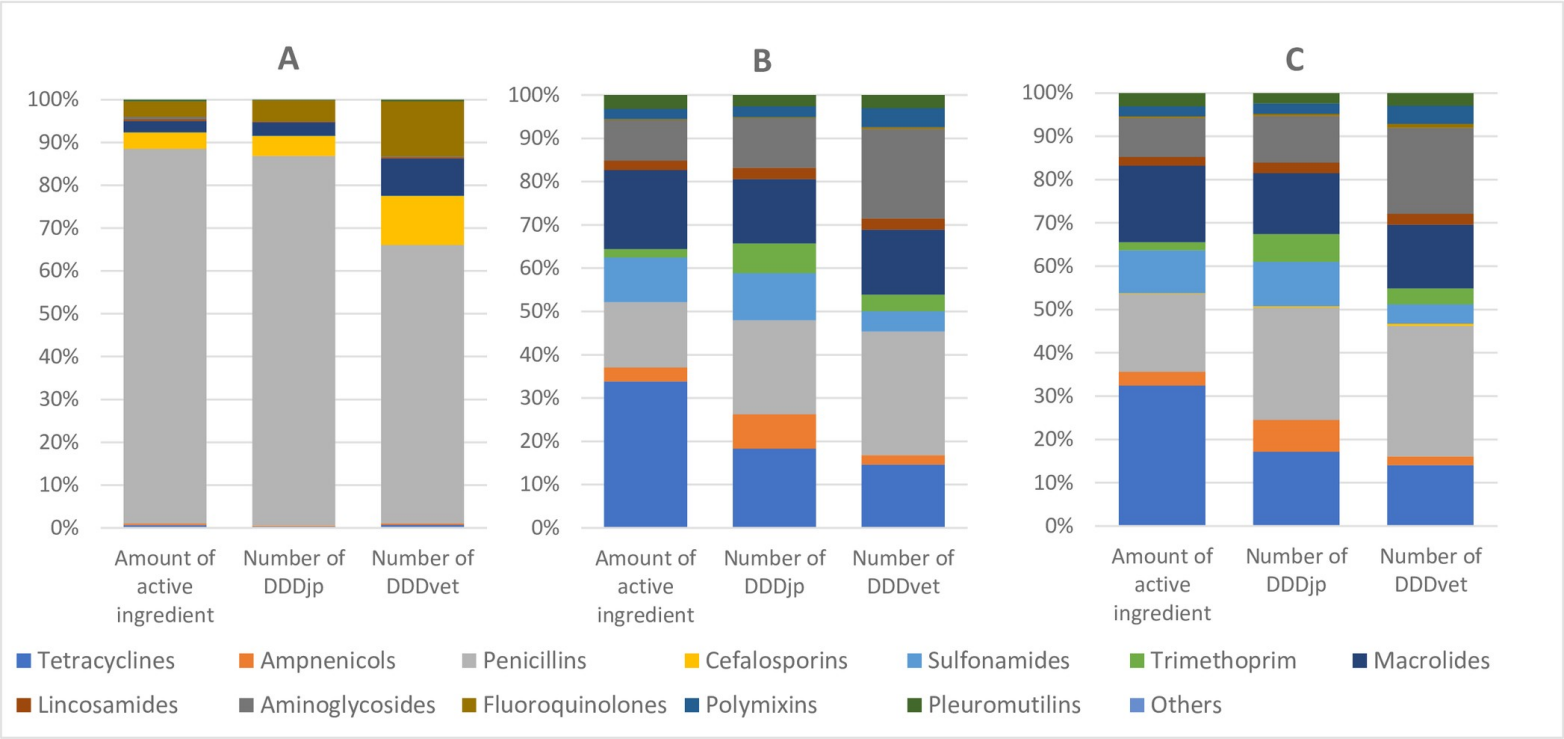
Relative distribution of antimicrobial use by administration route (injection (A), oral (B) and total (C)) between antimicrobial classes measured either as the weight of active ingredient or as the number of defined daily doses (DDDs), calculated using Japanese values (DDDjp) and European values (DDDvet) published by the European Medicine Agency (EMA).

The amount of active ingredient and the calculated numbers of DDDs for different antimicrobial classes are summarized in Table 3 and the relative distribution is presented in Fig 3. In terms of the total weight of active ingredient registered in this study, tetracyclines (32.5%) represented the largest proportion of total usage, followed by penicillins (18.0%), macrolides (17.6%) and sulfonamides (9.9%). Using DDDjp, penicillins (25.9%) represented the largest proportion of the total usage, followed by tetracyclines (17.1%), macrolides (14.1%) and aminoglycosides (10.7%). Using DDDvet, penicillins (30.1%) represented the largest proportion, followed by aminoglycosides (19.8%), macrolides (14.7%) and tetracyclines (14.0%).

**Table 3:** Total antimicrobial use on the 74 pig farms measured as active ingredient and by Japanese and European DDD grouped by different antimicrobial classes.

Antimicrobial class	Total amount of active ingredient in g			Number of DDDjp in 1000s			Number of DDDvet in 1000s		
	Injection	Oral	Total	Injection	Oral	Total	Injection	Oral	Total
Tetracyclines	5230 (0.6%)	6656500 (33.8%)	6661730 (32.5%)	805 (0.3%)	701641 (18.3%)	702445 (17.1%)	697 (0.7%)	310713 (14.6%)	311411 (14.0%)
Amphenicols	3000 (0.4%)	643600 (3.3%)	646600 (3.2%)	600 (0.2%)	303533 (7.9%)	304133 (7.4%)	316 (0.3%)	45146 (2.1%)	45462 (2.1%)
Penicillins	735820 (87.6%)	2964560 (15.1%)	3700380 (18.0%)	232674 (86.4%)	831042 (21.7%)	1063717 (25.9%)	61335 (65.0%)	607018 (28.6%)	668353 (30.1%)
Cephalosporins	31761 (3.8%)	0	31761 (0.2%)	12611 (4.7%)	0	12611 (0.3%)	10857 (11.5%)	0	10857 (0.5%)
Sulfonamides	700 (0.1%)	2035040 (10.3%)	2035740 (9.9%)	12 (0.0%)	417613 (10.9%)	417625 (10.2%)	16 (0.0%)	99661 (4.7%)	99677 (4.5%)
Trimethoprim	20 (0.0%)	381460 (1.9%)	381480 (1.9%)	3 (0.0%)	264214 (6.9%)	264218 (6.4%)	7 (0.0%)	81497 (3.8%)	81503 (3.7%)
Macrolides	22020 (2.6%)	3598650 (18.3%)	3620670 (17.6%)	8355 (3.1%)	568771 (14.9%)	577126 (14.1%)	8181 (8.7%)	318170 (15.0%)	326351 (14.7%)
Lincosamides	3140 (0.4%)	422160 (2.1%)	425300 (2.1%)	419 (0.2%)	99801 (2.6%)	100220 (2.4%)	314 (0.3%)	55547 (2.6%)	55861 (2.5%)
Aminoglycosides	4650 (0.6%)	1844803 (9.4%)	1849453 (9.0%)	310 (0.1%)	439290 (11.5%)	439600 (10.7%)	174 (0.2%)	438732 (20.7%)	438906 (19.8%)
Fluoroquinolones	29895 (3.6%)	47600 (0.2%)	77495 (0.4%)	13208 (4.9%)	8359 (0.2%)	21567 (0.5%)	12158 (12.9%)	8359 (0.4%)	20517 (0.9%)
Polymixins	0	467720 (2.4%)	467720 (2.3%)	0	97442 (2.5%)	97442 (2.4%)	0	93544 (4.4%)	93544 (4.2%)
Pleuromutilins	3940 (0.5%)	623853 (3.2%)	627793 (3.1%)	394 (0.1%)	98090 (2.6%)	98484 (2.4%)	328 (0.3%)	64315 (3.0%)	64643 (2.9%)
Others	0	0	0	0	0	0	0	0	0
Total	840176 (100%)	19685946 (100%)	20526121 (100%)	269391 (100%)	3829797 (100%)	4099188 (100%)	94383 (100%)	2122702 (100%)	2217085 (100%)

Of the injectable antimicrobials, penicillins represented the largest proportion (65.0–87.6%) regardless of the indicator used for calculation, followed by cephalosporins (3.8–11.5%) and fluoroquinolones (3.6–12.9%) (Fig 2A and Table 3). The relative distribution of injectable antimicrobials differed greatly depending on which of the DDDjp or DDDvet values were used.

Table 4 indicates the use of penicillin on the 74 study farms by different subclasses: beta-lactamase sensitive penicillins (procaine benzylpenicillin) and penicillins with extended spectrum (ampicillin and amoxicillin). Beta-lactamase sensitive penicillins are dominant in oral usage (88%) while penicillins with extended spectrum were dominant in injection (75%) in terms of the weight of active ingredient. In terms of the number of DDDs, penicillins with extended spectrum were dominant (75–88%) whether DDDjp or DDDvet were used.

**Table 4:** Total penicillin use on the 74 pig farms measured as active ingredient and by Japanese and European DDD grouped by different subclasses (beta-lactamase sensitive penicillins and penicillins with extended spectrum).

Antimicrobial subclass	Total amount of active ingredient in g			Number of DDDjp in 1000s			Number of DDDvet in 1000s		
	Injection	Oral	Total	Injection	Oral	Total	Injection	Oral	Total
Beta-lactamase sensitive penicillins	183910 (25%)	2596400 (88%)	2780310 (75%)	28291 (12%)	392757 (47%)	421048 (40%)	15330 (25%)	146818 (24%)	162148 (24%)
Penicillins with extended spectrum	551910 (75%)	368160 (12%)	920070 (25%)	204383 (88%)	438286 (53%)	642669 (60%)	46005 (75%)	460200 (76%)	506205 (76%)
Total penicilins	735820 (100%)	2964560 (100%)	3700380 (100%)	232674 (100%)	831042 (100%)	1063717 (100%)	61335 (100%)	607018 (100%)	668353 (100%)

### Antimicrobial use monitoring at farm level

Distribution of antimicrobial use by farm type and metric is shown in Table 5. The average antimicrobial use per farm in terms of DDDvets/PCU, DDDjps/PCU and mg of active ingredient/PCU were 36.3, 61.2 and 295.6 with standard deviation of 36.6, 53.5 and 270.7 respectively. There was a large variation of antimicrobial use at farm level with a right skewed distribution regardless of the metric used.

**Table 5:** Distribution of antimicrobial use on the 74 pig farms by farm type and metric.

Farm type	Metric	Average	SD	Minimum	25 percentile	Median	75 percentile	Maximum
All farms (n = 74)	DDDvets/PCU	36.3	36.6	0.1	9.9	22.7	59.0	153.5
	DDDjps/PCU	61.2	53.5	0.2	22.7	42.7	94.9	248.7
	mg active ingredient/PCU	295.6	270.7	1.6	111.3	225.5	391.3	1245.4
Farrowing farms (n = 15)	DDDvets/PCU	49.7	38.0	0.6	20.0	47.7	68.6	144.2
	DDDjps/PCU	70.4	45.6	1.7	36.3	67.9	98.7	160.5
	mg active ingredient/PCU	295.4	171.9	7.3	166.8	314.7	415.1	579.2
Finishing farms (n = 3)	DDDvets/PCU	12.9	11.4	1.3	7.2	13.2	18.6	24.1
	DDDjps/PCU	20.4	14.8	4.0	14.1	24.3	28.5	32.8
	mg active ingredient/PCU	148.6	116.7	17.4	102.3	187.2	214.2	241.1
Farrow-to- finish farms (n = 56)	DDDvets/PCU	34.0	36.3	0.1	9.4	17.0	57.6	153.5
	DDDjps/PCU	61.0	56.1	0.2	21.3	39.9	89.8	248.7
	mg active ingredient/PCU	303.5	296.6	1.6	109.5	210.6	396.5	1245.4

SD: Standard deviation.

**Fig 4:**
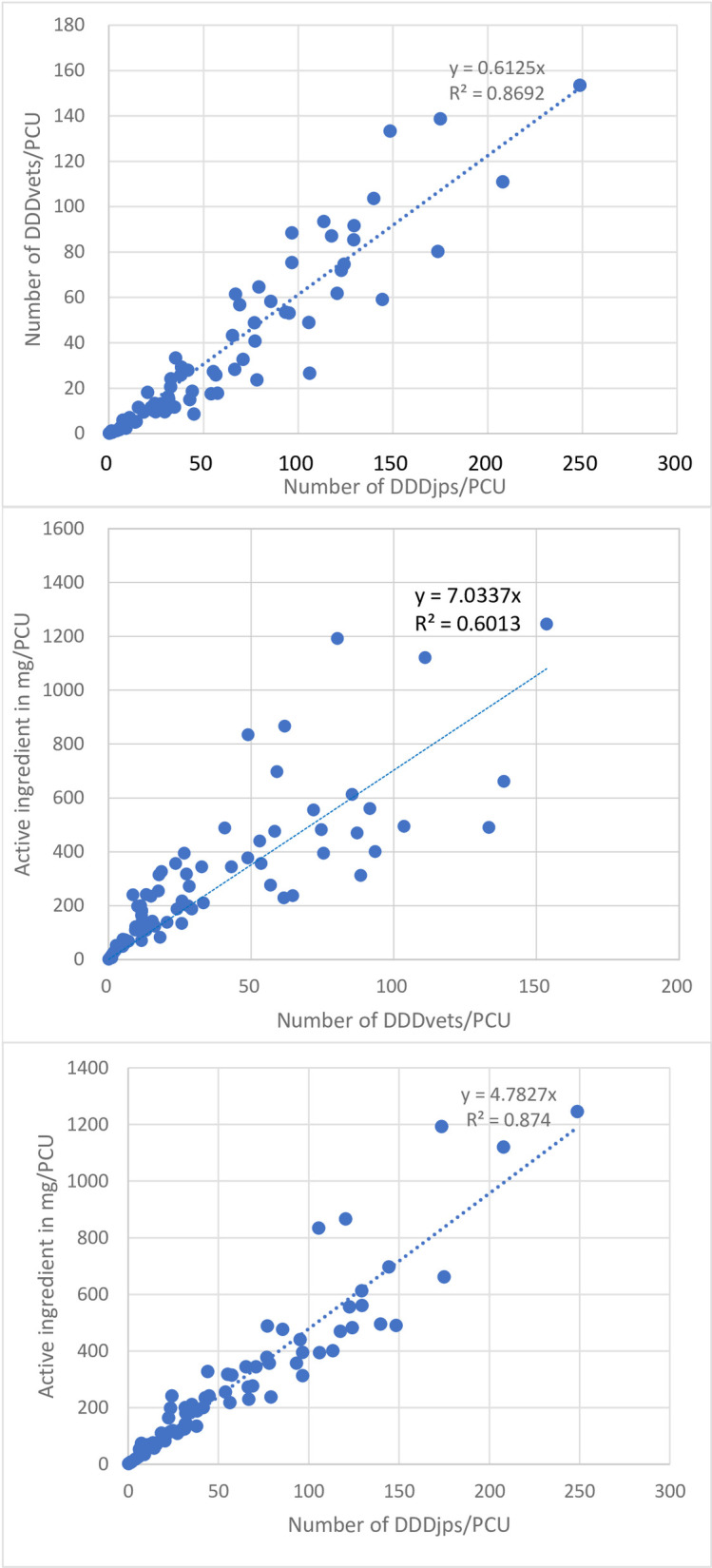
Scatterplots of weight of active ingredient and defined daily doses (DDD) per kg of PCU (population correction unit) at the farm level calculated using Japanese values (DDDjp) and European values (DDDvet). Each circle represents one farm (n = 74). After each dataset was tested for normality using the Shapiro-Wilk test with the null hypothesis for all datasets being declined, correlation was investigated by Spearman’s Rho test and found to be significantly correlated (*P*<0.001).

The scatterplot of calculated numbers of DDDs per kg of PCU on the 74 farms which enables a visual analysis of the association between Japanese (jp) and European (vet) definitions is given in Fig 4. As shown, both the calculated numbers of DDDs revealed a positive correlation between results on the farm level by Spearman’s rank correlation test, with Rho values of 0.950 (*P*<0.01) between the numbers of DDDjps and DDDvets, and 0.968 (*P*<0.01) between the number of DDDjps and the weight of active ingredient.

**Fig 5:**
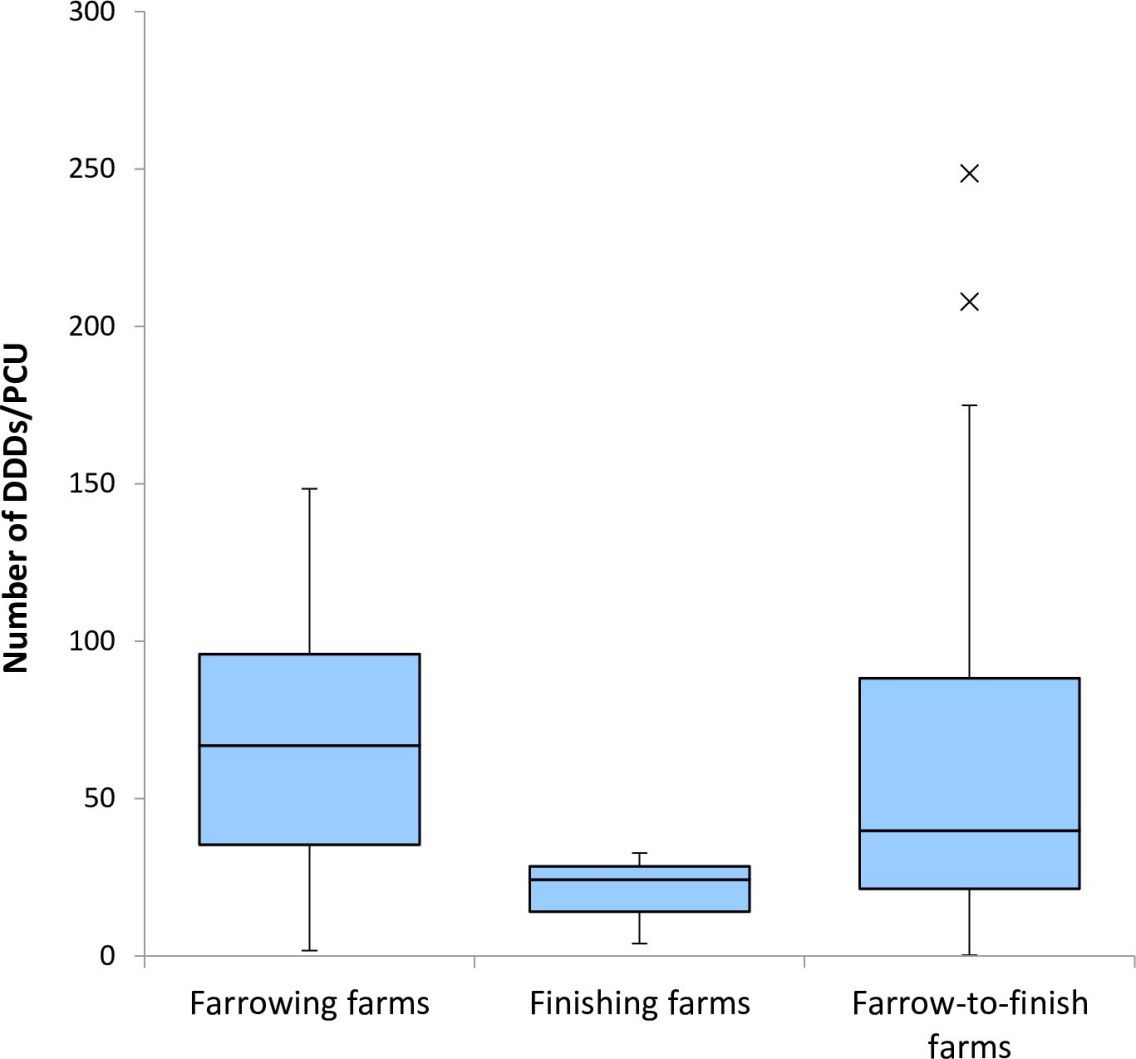
Box-and-whisker plot of antimicrobial use in the number of defined daily doses (DDDjp) per kg of PCU (population correction unit) on different types of farms (farrowing, finishing and farrow-to-finish farms) in 2019. The dots represent the range out of ±1.5×interquartile range (IQR). No significant difference was observed between any types of farms as a result of Kruskul-Wallis test (*P* = 0.19).

After the null hypothesis was rejected for normality of all datasets, the Kruskal-Wallis test revealed no significant difference in antimicrobial use between any two types of farrowing farms, finishing farms and farrow-to-finish farms (*P* = 0.19) (Fig 5). Distributions of DDDjps/PCU, DDDvets/PCU and weight of active ingredient/PCU per farm for these three types of farms are presented in Table 5.

## Discussion

This study is the first attempt to measure antimicrobial use on pig farms in Japan at farm level using defined daily doses (DDDs). It reveals that whether Japanese or European DDD values are used, the antimicrobial use in terms of the number of DDDs and weight of active ingredient provide similar results with a positive correlated association. However, differences still remain, namely, in the evaluation of the different active ingredient classes and different administration routes (Tables 2 and 3 and Figs 2 and 3). In terms of the weight of active ingredient, tetracyclines represented the largest proportion of total usage, followed by penicillins and macrolides, whereas in terms of the number of DDDs using Japanese DDD values penicillins were most frequently used, followed by tetracyclines and macrolides. Using DDDvet, penicillins were most frequently used followed by aminoglycosides and macrolides. This indicates that use of an indicator based on DDD, in particular one based on Japanese DDD values (DDDjp) which offers a more accurate reflection of the antimicrobial selection pressure [10], is essential when monitoring antimicrobial use in Japan.

In terms of the relative distribution by administration route, our study revealed that a large proportion (93.4–95.9%) of antimicrobial use was by oral administration. This is lower than the proportion (96.5–97.9%) observed in our previous study using national sales data [26], but is higher than in European countries, where the average proportion in terms of weight of active ingredient per PCU was 87.7% in 2018 [9]. This indicates that even in those 74 pig farms under the consultation of JASV veterinarians, a large proportion of antimicrobials are used for prophylactic and metaphylactic purposes.

Quantification of penicillin use by different subclasses revealed that in terms of weight of active ingredient, beta-lactamase sensitive penicillins represented 75% of the total penicillin use. This proportion is relatively high compared with Europe, where the use of beta-lactamase sensitive penicillins in Finland, Iceland, Norway and Sweden only exceeds 75% of the total penicillin use [9]. Also, our result revealed that the relative distribution of these two subclasses reversed when the dosage-based indicator was used. This suggests the use of a dosage-based indicator is essential when monitoring antimicrobial use.

It is also remarkable that the number of DDDjps of injectable antimicrobials deviates more from the corresponding number of DDDvets, compared to orally administered antimicrobials. This is because the DDDjp value of procaine benzylpenicillin (2.7mg/kg), the most commonly used injectable antimicrobial is much smaller than the corresponding European DDDvet value (13.0mg/kg). The first injectable procaine benzylpenicillin products were approved in Japan in 1963 with this small dosage. The new products containing this active ingredient subsequently approved have been approved with the same dosage, and this resulted in assigning this active ingredient this small DDDjp value compared to the corresponding DDDvet value. Further investigations are needed to verify if this is a case where an outdated dosages is used without reflecting the risk of selection or emergence of resistance bacteria.

Our results also revealed that antimicrobial use monitoring systems at farm level will provide similar results in identification of heavy antimicrobial users regardless of whether the Japanese or European DDD value is used (Fig 3). Surprisingly, associations between the numbers of DDDvets and DDDjps and between the number of DDDjps and weight of active ingredients showed almost equal correlation with Rho values of 0.951 and 0.957, respectively (Fig 2). Echtermann *et al*. (2019), using Swiss DDDs and Swiss pig farm data, compared DDDvets/pig and DDDchs/pig at the farm level and reported similar results with Rho values (0.968–0.976) [27]. O’Neil *et al*. (2020) compared antimicrobial use at farm level measured using Dutch, Danish and German dosage-based indicators and weight of active ingredient and much lower Rho values (0.76–0.92) were obtained [28]. This can probably be explained by the fact that the latter used not only different DDD values but also different standard weights of sows, weaners and fattening pigs in calculating the Dutch, Danish and German indicators.

Our results also revealed that the number of DDDjps/PCU was greater than the number of DDDvets/PCU for all 74 farms analyzed in this study (Fig 3). This was attributed to the fact that DDDjp values are lower than DDDvet values for most antimicrobial agents [19]. Also, this study observed that DDDvets did not cover all the antimicrobial agents used in veterinary medicine in Japan. The absence of DDDvet values for some antimicrobial agents used in Japan and the differences in the number of DDDjps/PCU and DDDvets/PCU appear to confirm the need for Japanese DDDs, which better reflect antimicrobial selection pressure in a Japanese context.

Our results revealed a significant variation of antimicrobial use at farm level between farms, with a right skewed distribution with several heavy users affecting the mean value (Figs 4 and 5). This was observed in our previous study using an indicator based on weight of active ingredient [29] and in previous studies in other countries, such as Belgium, Denmark, the Netherlands, Germany and Italy [30–33]. This confirms that a successful reduction in antimicrobial usage can be achieved by subjecting heavy antimicrobial users to intensive reduction measures.

Our results indicate that the pig farms included in the analysis were using 295mg of active ingredient per kg PCU per farm (Table 5), which is 32% less antimicrobials than the amount estimated based on sales data obtained from market authorization holders (437 mg of active ingredient per kg PCU in 2017) [34]. Also, the proportion of antimicrobial use by oral administration is lower on these farms (93.4–95.9%) than the national average calculated using sales data (96.5–97.9%) [26]. These are most likely because under JASV member veterinarians’ consultation, the farms in this study revealed a higher level of awareness in regard to antimicrobial use than on other pig farms in Japan. (Mandatory periodical inspections of pig farms by veterinarians for herd health management have been introduced since July 2020 under the Domestic Animal Infectious Diseases Control Law [35]). In addition to the awareness level, the farms analyzed in this study are not geographically evenly distributed (Fig 1 and Table 1). Therefore, our results might not be representative of the pig population across Japan. Further studies are needed with more representative data to verify if the antimicrobial use at farm level matches the national consumption data. Nevertheless, it provides useful information as to the adequacy of using Japanese DDD values in monitoring antimicrobial use at farm level.

Previous studies have revealed that antimicrobial consumption differs between production stages with weaners being the heaviest antimicrobial consumers and fattening pigs the lowest antimicrobial consumers) [33,36–40]. Our results obtained from the different farm types indicate that farrowing farms and farrow-to-finish farms tend to have higher antimicrobial use than finishing farms. However, no significant difference (*P* = 0.19) was observed between any two types of farms (Fig 5). This is most likely because of the insufficient statistical power due to the small sample size of study farms, in particular of farrowing farms (n = 3).

Most European countries are moving toward a more responsible use of antimicrobials with 25 countries seeing overall sales falling from 161.4 mg/PCU in 2011 to 105.6 mg/PCU in 2018 (reduction of 34.6%) [9]. The authors have previously investigated the use of antimicrobial agents in food-producing animals in Japan and revealed that annual use of veterinary antimicrobials in food-producing animals in Japan remained 203–229 mg of active ingredient per kg of PCU between 2014 and 2017 [7,34,41], which is relatively high compared with the usage in most European countries. Development of dosage-based indicators using Japanese DDD values such as the one used in this study will help monitor the antimicrobial use in Japan more accurately as they reflect the selection pressure taking account of potencies of different antimicrobial agents.

## Supporting information

S1 Table Japanese DDD values (DDDjp) defined in this study for antimicrobial agents used in pigs in Japan and corresponding DDD values (DDDvet) defined by the European Medicines Agency stratified by unique combination of administration route and active ingredient. (DOCX)
