# Supplementary material for: Antimicrobial use on 74 Japanese pig farms in 2019: A comparison of Japanese and European defined daily doses in the field
Source: PLoS One. 2021 Aug 6;16(8):e0255632. doi: 10.1371/journal.pone.0255632 (PMC8345878; doi:10.1371/journal.pone.0255632)
Supplement: S1 Table — (DOCX) [file pone.0255632.s001.docx]

Table S1

Japanese DDD values (DDDjp) defined in this study for antimicrobial agents used in pigs in Japan and corresponding DDD values (DDDvet) defined by the European Medicines Agency stratified by unique combination of administration route and active ingredient

| Antimicrobial class | Antimicrobial agent  (active ingredient) | Product type | Administration route | DDDvet  (mg/kg) | DDDjp  (mg/kg) |
| --- | --- | --- | --- | --- | --- |
| Tetracyclines | Oxytetracycline | Single substance | Injection | 7.5 | 6.5 |
| Amphenicols | Thiamphenicol | Single substance | Injection | 75.0 | 20.0 |
|  | Florfenicol | Single substance | Injection | 9.5 | 5.0 |
| Penicillins | Ampicillin | Single substance | Injection | 12.0 | 6.5 |
|  | Amoxicillin | Single substance | Injection | 8.9 | 7.5 |
|  | Procaine Benzylpenicillin | Single substance | Injection | 13.0 | 2.7 |
|  | Procaine Benzylpenicillin | Combination | Injection |  | 7.2 |
| Cefalosporins | Ceftiofur | Single substance | Injection | 3.0 | 2.6 |
|  | Cefquinome | Single substance | Injection | 1.9 | 1.5 |
| Sulfonamides | Sulfadimethoxine | Single substance | Injection | 30.0 | 60.0 |
|  | Sulfamonomethoxine | Single substance | Injection |  | 70.0 |
|  | Sulfadoxine | Combination | Injection | 14.0 | 30.0 |
| Trimethoprim | Trimethoprim | Combination | Injection | 3.0 | 6.0 |
| Macrolides | Tylosin | Single substance | Injection | 13.0 | 6.0 |
|  | Tulathromycin | Single substance | Injection |  | 2.5 |
| Lincosamides | Lincomycin | Single substance | Injection | 10.0 | 7.5 |
| Aminoglycosides | Dihydrostreptomycin | Single substance | Injection | 20.0 | 60.0 |
|  | Dihydrostreptomycin | Combination | Injection |  | 15.0 |
|  | Kanamycin | Single substance | Injection | 28.0 | 15.0 |
|  | Kanamycin | Single substance | Intranasal |  | 5.2 |
| Quinolones | Enrofloxacin | Single substance | Injection | 3.4 | 2.6 |
|  | Danofloxacin | Single substance | Injection | 1.2 | 1.3 |
|  | Marbofloxacin | Single substance | Injection | 2.0 | 2.0 |
|  | Orbifloxacin | Single substance | Injection |  | 3.8 |
| Pleuromutilins | Tiamulin | Single substance | Injection | 12.0 | 10.0 |
| Tetracyclines | Doxycycline | Single substance | Oral | 11.0 | 9.0 |
|  | Chlortetracycline | Single substance | Oral | 31.0 | 10.8 |
|  | Chlortetracycline | Combination | Oral |  | 6.0 |
|  | Oxytetracycline | Single substance | Oral | 26.0 | 9.4 |
|  | Oxytetracycline | Combination | Oral |  | 7.0 |
| Amphenicoles | Thianphenicol | Single substance | Oral | 35.0 | 5.0 |
|  | Florfenicol | Single substance | Oral | 10.0 | 1.5 |
| Penicillins | Ampicillin | Single substance | Oral | 30.0 | 8.0 |
|  | Amoxicillin | Single substance | Oral | 17.0 | 6.5 |
|  | Procain benzylpenicillin | Combination | Oral |  | 0.8 |
| Sulfonamides | Sulfadimethoxine | Single substance | Oral | 48.0 | 54.0 |
|  | Sulfadimethoxine | Combination | Oral | 24.0 | 28.8 |
|  | Sulfamonomethoxine | Single substance | Oral |  | 40.0 |
|  | Sulfamonomethoxine | Combination | Oral | 9.4 | 8.6 |
|  | Sulfamethoxazole | Combination | Oral | 20.0 | 4.7 |
|  | Sulfadimidine | Combination | Oral | 23.0 | 6.0 |
| Trimethoprims | Trimethoprim | Combination | Oral | 4.7 | 1.4 |
|  | Ormethoprim | Combination | Oral |  | 2.9 |
| Macrolides | Tylosin | Single substance | Oral | 12.0 | 11.3 |
|  | Tilmicosin | Single substance | Oral | 15.0 | 5.0 |
|  | Tylvalosin | Single substance | Oral | 3.6 | 1.4 |
|  | Mirosamycin | Single substance | Oral |  | 2.5 |
| Lincosamides | Lincomycin | Single substance | Oral | 7.6 | 4.2 |
| Aminoglycosides | Streptomycin | Single substance | Oral |  | 20.0 |
|  | Streptomycin | Combination | Oral |  | 4.2 |
|  | Gentamicin | Single substance | Oral | 1.4 | 0.6 |
|  | Kanamycin | Combination | Oral |  | 4.2 |
|  | Apramycin | Single substance | Oral | 9.0 | 4.0 |
|  | Fragiomycin | Combination | Oral |  | 4.9 |
| Fluoroquinolones | Norfloxacin | Single substance | Oral |  | 7.5 |
|  | Orbifloxacin | Single substance | Oral |  | 3.80 |
| Other quinolones | Oxolinic acid | Single substance | Oral | 26.0 | 20.0 |
|  | Tiamulin | Single substance | Oral | 9.7 | 6.4 |
| Pleuromutilins | Valnemulin | Single substance | Oral | 5.3 | 2.6 |
| Polymyxins | Colistin | Single substance | Oral | 5.0 | 4.8 |

DDDvet DDD values in mg/kg/day defined by the European Medicines Agency (EMA)

DDDjp DDD values in mg/kg/day defined in this study using DDD values of antimicrobial products approved and marketed for use in Japan

Source Fujimoto K, Kawasaki M, Abe R, Yokoyama T, Haga T, Sugiura K. Establishing defined daily doses (DDDs) for antimicrobial agents used in pigs, cattle and poultry in Japan and comparing them with European DDD values. Plos ONE (in press) [Ref. No. 19]
